# Supplementary material for: Limitations and Modifications of Skin Sensitization NAMs for Testing Inorganic Nanomaterials
Source: Toxics. 2024 Aug 21;12(8):616. doi: 10.3390/toxics12080616 (PMC11360696; doi:10.3390/toxics12080616)
Supplement: Supplementary file 1 [file toxics-12-00616-s001.zip › toxics-3140318-supplementary.pdf]

(1) CeO2 (NM 212)

Results of DPRA with CeO2 (NM 212)

TableS1a: DPRA with CeO2 (NM 212). Peak area, peptide concentration and peptide depletion of NC, PC and the test substance for cysteine-peptide.

	peak area [mAU*s] at 220 nm			peptide concentration [mM]				
**Reaction with cysteine-peptide**	**sample 1**	**sample 2**	**sample 3**	**sample 1**	**sample 2**	**sample 3**	**mean**	**SD**
**NC: 0.05% BSA**	464.9	461.3	461.4	0.497	0.493	0.493	0.494	0.002
**CeO2 (NM 212) (ratio to peptide 1.5 : 1)**	442.5	450.1	449.8	0.473	0.481	0.481	0.478	0.005
**PC: EGDMA in 0.05% w/v BSA water**	85.9	111.5	107.1	0.090	0.117	0.113	0.107	0.015

	peptide depletion [%]				
**Reaction with cysteine-peptide**	**sample 1**	**sample 2**	**sample 3**	**mean**	**SD**
**NC: 0.05% BSA**	-0.52	0.28	0.24	**0.00**	0.45
**CeO2 (NM 212) (ratio to peptide 1.5 : 1)**	4.37	2.71	2.76	**3.28**	0.94
**PC: EGDMA in 0.05% w/v BSA water**	81.83	76.25	77.22	**78.43**	2.98

TableS1b: DPRA with CeO2 (NM 212). Peak area, peptide concentration and peptide depletion of NC, PC and the test substance for lysine-peptide.

	peak area [mAU*s] at 220 nm			peptide concentration [mM]				
**Reaction with lysine-peptide**	**sample 1**	**sample 2**	**sample 3**	**sample 1**	**sample 2**	**sample 3**	**mean**	**SD**
**NC: 0.05% BSA**	453.2	452.4	452.9	0.511	0.510	0.511	0.511	0.000
**CeO2 (NM 212) (ratio to peptide 7.4 : 1)**	452.5	448.8	449.1	0.510	0.506	0.507	0.508	0.002
**PC: EGDMA in 0.05% w/v BSA water**	406.3	401.3	405.2	0.458	0.452	0.457	0.456	0.003

	peptide depletion [%]				
**Reaction with lysine-peptide**	**sample 1**	**sample 2**	**sample 3**	**mean**	**SD**
**NC: 0.05% BSA**	-0.08	0.10	-0.02	**0.00**	0.09
**CeO2 (NM 212) (ratio to peptide 7.4 : 1)**	0.08	0.90	0.84	**0.60**	0.46
**PC: EGDMA in 0.05% w/v BSA water**	10.37	11.47	10.61	**10.81**	0.58

Mean peptide depletion

TableS1c: DPRA with CeO2 (NM 212). Mean peptide depletions of cysteine, lysine and both peptides.

	Cysteine-Peptide		Lysine-Peptide		
	mean depletion [%]	SD [%]	mean depletion [%]	SD [%]	mean of both depletions [%]
**CeO2 (NM 212) (ratio to peptide 1.5 : 1 or 7.4 : 1)**	3.28	0.94	0.60	0.46	**1.94**
**PC: EGDMA in 0.05% w/v BSA water**	78.43	2.98	10.81	0.58	**44.62**

Results of LuSens with CeO2 (NM 212)

Preliminary cytotoxicity assessment

TableS2a: LuSens with CeO2 (NM 212). Results of preliminary cytotoxicity assessment using the MTT assay. Final test substance concentrations were calculated considering the purity/contents of 99%. The CV75 value (= estimated concentration that affords 75% cell viability) was determined by linear regression from the concentration-response curve of the MTT pre-test to be 532 µg/mL (corresponding to ca. 3093 µM). However, in order to achieve relevant cytotoxicity (< 70% relative viability), the top concentration for the main experiment was chosen to be 3718 µM.

Concentration (final test substance) [µM]	Concentration (final test substance) [µg/mL]	Concentration (test substance) [µM]	Concentration (test substance) [µg/mL]	mean $OD_{570-690}$ of 3 replicates	mean rel. viability [%]
VC	VC	**VC**	**VC**	0.170	**100**
0.92	0.16	**0.93**	**0.16**	0.133	**78**
1.8	0.32	**1.9**	**0.32**	0.128	**75**
9.2	1.6	**9.3**	**1.6**	0.132	**78**
18	3.2	**19**	**3.2**	0.132	**78**
92	16	**93**	**16**	0.141	**83**
184	32	**186**	**32**	0.132	**78**
920	158	**930**	**160**	0.143	**85**
1841	317	**1859**	**320**	0.160	**95**
3681	634	**3718**	**640**	0.110	65

Main experiments

TableS2b: LuSens with CeO2 (NM 212). Mean values and standard deviations of luciferase induction and rel. viability as well as p-values of t-test (experiment 1). Concentrations with fold inductions above 1.50 with rel. viability ≥70% and with statistical significance are indicated in bold and in grey when < 70% viability.

Concentration (test substance) [µg/mL]	Concentration (test substance) [µM]	1st experiment fold induction mean	SD	rel. viability [%] mean	SD	t-test p-value	markers
179	1038	0.36	0.03	112	3	0.000	**
214	1245	0.31	0.01	107	3	0.000	**
257	1494	0.38	0.01	111	2	0.000	**
309	1793	0.37	0.05	111	2	0.000	**
370	2152	0.39	0.07	104	8	0.000	**
444	2582	0.35	0.05	102	12	0.000	**
533	3098	0.35	0.00	103	11	0.000	**
640	3718	0.30	0.05	107	3	0.000	**
VC	VC	1.00	0.11	100	10	-	-
**EGDMA 18 µg/mL**	**EGDMA 90.8 µM**	**4.18**	**0.37**	**81**	**7**	**0.000**	**
LA 450 µg/mL	LA 5000 µM	0.88	0.03	106	6	0.000	**

TableS2c: LuSens with CeO2 (NM 212). Mean values and standard deviations of luciferase induction and rel. viability as well as p-values of t-test (experiment 2, centrifugation step before MTT measurement). Concentrations with fold inductions above 1.50 with rel. viability ≥70% and with statistical significance are indicated in bold and in grey when < 70% viability.

Concentration (test substance) [µg/mL]	Concentration (test substance) [µM]	2nd experiment, centrifuged fold induction mean	SD	rel. viability [%] mean	SD	t-test p-value	markers
179	1038	0.57	0.12	98	4	0.007	**
214	1245	0.50	0.03	65	3	0.000	**
257	1494	0.71	0.12	84	3	0.019	*
309	1793	0.64	0.17	83	4	0.030	*
370	2152	0.44	0.06	82	4	0.000	**
444	2582	0.48	0.05	77	7	0.000	**
533	3098	0.47	0.04	73	5	0.000	**
640	3718	0.30	0.21	69	4	0.011	*
VC	VC	1.00	0.12	100	12	-	-
**EGDMA 18 µg/mL**	**EGDMA 90.8 µM**	**7.67**	**1.98**	**96**	**19**	**0.001**	**
LA 450 µg/mL	LA 5000 µM	1.08	0.10	105	3	0.082	n.s.

TableS2d: LuSens with CeO2 (NM 212). Mean values and standard deviations of luciferase induction and rel. viability as well as p-values of t-test (experiment 3, centrifugation step before MTT measurement). Concentrations with fold inductions above 1.50 with rel. viability ≥70% and with statistical significance are indicated in bold and in grey when < 70% viability.

Concentration (test substance) [µg/mL]	Concentration (test substance) [µM]	3rd experiment, centrifuged fold induction mean	SD	rel. viability [%] mean	SD	t-test p-value	markers
179	1038	0.44	0.05	102	6	0.000	**
214	1245	0.47	0.05	86	2	0.000	**
257	1494	0.37	0.04	89	5	0.000	**
309	1793	0.37	0.04	90	2	0.000	**
370	2152	0.36	0.06	84	3	0.000	**
444	2582	0.37	0.02	84	1	0.000	**
533	3098	0.34	0.02	81	3	0.000	**
640	3718	0.44	0.06	81	6	0.000	**
VC	VC	1.00	0.10	100	4	-	-
**EGDMA 18 µg/mL**	**EGDMA 90.8 µM**	**3.74**	**0.20**	**87**	**4**	**0.000**	**
LA 450 µg/mL	LA 5000 µM	0.90	0.18	102	4	0.131	n.s.

Results of h-CLAT with CeO2 (NM 212)

Main experiments

TableS3a: h-CLAT with CeO2 (NM 212). RFI CD86, RFI CD54 and rel. viability. Mean values and standard deviations of 1st experiment with DGC. RFI above 150% (CD86) or 200% (CD54) with rel. viability ≥50% are indicated in bold.

Concentration (test substance) [µg/mL]	1st experiment RFI CD86 mean [%]	RFI CD86 SD [%]	RFI CD54 mean [%]	RFI CD54 SD [%]	Viability rel. viability [%]	SD of viability
**357**	104	1	82	2	**80**	2
**429**	105	2	98	3	**78**	4
**514**	109	4	78	4	**72**	2
**617**	109	6	78	4	**71**	4
**741**	111	6	74	14	**58**	1
**889**	104	13	70	8	**58**	2
**1067**	101	5	41	10	48	2
**1280**	102	7	86	20	**50**	2
**VC**	100	21	100	18	**100**	1
**LA 1000 µg/mL**	70	3	108	16	**100**	0
**DNCB 4 µg/mL**	**349**	32	**579**	104	**82**	8

TableS3b: h-CLAT with CeO2 (NM 212). RFI CD86, RFI CD54 and rel. viability. Mean values and standard deviations of 2nd experiment with DGC. RFI above 150% (CD86) or 200% (CD54) with rel. viability ≥50% are indicated in bold.

Concentration (test substance) [µg/mL]	2nd experiment RFI CD86 mean [%]	RFI CD86 SD [%]	RFI CD54 mean [%]	RFI CD54 SD [%]	Viability rel. viability [%]	SD of viability
**357**	118	11	100	7	**68**	4
**429**	123	15	90	7	**69**	2
**514**	140	19	86	27	**59**	4
**617**	133	9	90	7	**50**	3
**741**	127	23	48	0	42	4
**889**	139	34	43	61	38	6
**1067**	104	0	-29	40	34	2
**1280**	120	0	24	34	33	4
**VC**	100	28	100	12	**100**	0
**LA 1000 µg/mL**	63	9	114	13	100	0
**DNCB 4 µg/mL**	**389**	21	**583**	103	**81**	3

(2) BaSO4 (NM 220)

Results of DPRA with BaSO4

TableS4a: DPRA with BaSO4. Peak area, peptide concentration and peptide depletion of NC, PC and the test substance for cysteine-peptide.

Reaction with cysteine-peptide	peak area [mAU*s] at 220 nm sample 1	sample 2	sample 3	peptide concentration [mM] sample 1	sample 2	sample 3	mean	SD
**NC: 0.05% BSA**	492.2	492.4	494.0	0.508	0.509	0.510	0.509	0.001
**BaSO4 (ratio to peptide 1.0 : 1)**	487.7	479.8	482.2	0.504	0.496	0.498	0.499	0.004
**PC: EGDMA in 0.05% w/v BSA water**	93.0	87.5	81.0	0.094	0.088	0.081	0.088	0.006

Reaction with cysteine-peptide	peptide depletion [%] sample 1	sample 2	sample 3	mean	SD
**NC: 0.05% BSA**	0.14	0.10	-0.24	**0.00**	0.21
**BaSO4 (ratio to peptide 1.0 : 1)**	1.05	2.65	2.18	**1.96**	0.82
**PC: EGDMA in 0.05% w/v BSA water**	81.57	82.69	84.00	**82.75**	1.22

TableS4b: DPRA with BaSO4. Peak area, peptide concentration and peptide depletion of NC, PC and the test substance for lysine-peptide.

Reaction with lysine-peptide	peak area [mAU*s] at 220 nm			peptide concentration [mM]				
	sample 1	sample 2	sample 3	sample 1	sample 2	sample 3	mean	SD
NC: 0.05% BSA	435.4	428.0	433.7	0.508	0.499	0.506	0.505	0.005
BaSO4 (ratio to peptide 5.2 : 1)	431.7	433.5	431.8	0.504	0.506	0.504	0.505	0.001
PC: EGDMA in 0.05% w/v BSA water	383.6	374.3	381.4	0.447	0.436	0.445	0.443	0.006

Reaction with lysine-peptide	peptide depletion [%]				
	sample 1	sample 2	sample 3	mean	SD
NC: 0.05% BSA	-0.72	1.02	-0.30	**0.00**	0.91
BaSO4 (ratio to peptide 5.2 : 1)	0.16	-0.27	0.13	**0.00**	0.24
PC: EGDMA in 0.05% w/v BSA water	11.35	13.50	11.86	**12.24**	1.13

Mean peptide depletion

TableS4c: DPRA with BaSO4. Mean peptide depletions of Cysteine, Lysine and both peptides.

	Cysteine-Peptide		Lysine-Peptide		
	mean depletion [%]	SD [%]	mean depletion [%]	SD [%]	mean of both depletions [%]
BaSO4 (ratio to peptide 1.0 : 1 or 5.2 : 1)	1.96	0.82	0.00	0.24	**0.98**
PC: EGDMA in 0.05% w/v BSA water	82.75	1.22	12.24	1.13	**47.49**

Results of LuSens with BaSO4

Preliminary cytotoxicity assessment

TableS5a: LuSens with BaSO4. Results of preliminary cytotoxicity assessment using the MTT assay. Final test substance concentrations were calculated considering the purity/contents of 95%.

Concentration (final test substance) [µM]	Concentration (final test substance) [µg/mL]	Concentration (test substance) [µM]	Concentration (test substance) [µg/mL]	mean $OD_{570-690}$ of 3 replicates	mean rel. viability [%]
VC	VC	VC	VC	0.297	**100**
0.7	0.2	0.7	0.2	0.250	**84**
1.3	0.3	1.4	0.3	0.238	**80**
6.5	1.5	6.9	1.6	0.247	**83**
13	3.0	14	3.2	0.251	**84**
65	15	69	16	0.277	**93**
130	30	137	32	0.240	**81**
651	152	686	160	0.266	**89**
1303	304	1371	320	0.303	**102**
2605	608	2742	640	0.237	**80**

Main experiments

TableS5b: LuSens with BaSO4. Mean values and standard deviations of luciferase induction and rel. viability as well as p-values of t-test (experiment 2). Concentrations with fold inductions above 1.50 with rel. viability ≥70% and with statistical significance are indicated in bold and in grey when < 70% viability.

Concentration (test substance) [µg/mL]	Concentration (test substance) [µM]	2nd experiment fold induction mean	SD	rel. viability [%] mean	SD	t-test p-value	markers
179	765	1.29	0.07	124	10	0.005	**
214	918	1.29	0.11	102	6	0.019	*
257	1102	1.19	0.12	116	4	0.050	*
309	1322	1.13	0.06	112	5	0.024	*
370	1587	1.05	0.03	117	1	0.048	*
444	1904	0.98	0.18	106	1	0.428	n.s.
533	2285	0.92	0.06	99	2	0.071	n.s.
640	2742	0.87	0.15	85	3	0.145	n.s.
VC	VC	1.00	0.07	100	10	-	-
**EGDMA 18 µg/mL**	**EGDMA 90.8 µM**	**8.04**	**0.29**	**109**	**13**	**0.000**	**
LA 450 µg/mL	LA 5000 µM	1.07	0.14	104	7	0.178	n.s.

TableS5c: LuSens with BaSO4. Mean values and standard deviations of luciferase induction and rel. viability as well as p-values of t-test (experiment 3). Concentrations with fold inductions above 1.50 with rel. viability ≥70% and with statistical significance are indicated in bold and in grey when < 70% viability.

Concentration (test substance) [µg/mL]	Concentration (test substance) [µM]	3rd experiment fold induction mean	SD	rel. viability [%] mean	SD	t-test p-value	markers
179	765	1.41	0.22	122	3	0.038	*
214	918	1.14	0.11	96	3	0.076	n.s.
257	1102	1.19	0.14	108	4	0.059	n.s.
309	1322	1.04	0.03	97	7	0.132	n.s.
370	1587	1.03	0.07	105	4	0.281	n.s.
444	1904	1.05	0.08	96	4	0.223	n.s.
533	2285	1.12	0.11	92	4	0.095	n.s.
640	2742	1.02	0.07	91	5	0.390	n.s.
VC	VC	1.00	0.13	100	6	-	-
**EGDMA 18 µg/mL**	**EGDMA 90.8 µM**	**5.42**	**0.55**	**80**	**5**	**0.000**	**
LA 450 µg/mL	LA 5000 µM	0.86	0.06	109	12	0.001	**

Results of h-CLAT with BaSO4

Main experiments

TableS6a: h-CLAT with BaSO4. RFI CD86, RFI CD54 and rel. viability. Mean values and standard deviations of 2nd experiment with DGC. RFI above 150% (CD86) or 200% (CD54) with rel. viability ≥50% are indicated in bold.

Concentration (test substance) [µg/mL]	2nd experiment RFI CD86 mean [%]	RFI CD86 SD [%]	RFI CD54 mean [%]	RFI CD54 SD [%]	Viability rel. viability [%]	Viability SD of viability
**357**	95	15	89	9	**96**	1
**429**	104	28	84	16	**93**	1
**514**	100	9	84	3	**91**	1
**617**	106	12	79	9	**93**	2
**741**	101	38	70	9	**91**	1
**889**	85	-	70	9	**89**	3
**1067**	99	25	56	2	**84**	3
**1280**	94	9	65	15	**83**	3
**VC**	100	17	100	8	**100**	1
**LA 1000 µg/mL**	69	8	107	8	**101**	0
**DNCB 4 µg/mL**	**308**	13	**429**	25	**77**	6

TableS6b: h-CLAT with BaSO4. RFI CD86, RFI CD54 and rel. viability. Mean values and standard deviations of 3rd experiment with DGC. RFI above 150% (CD86) or 200% (CD54) with rel. viability ≥50% are indicated in bold.

Concentration (test substance) [µg/mL]	3rd experiment RFI CD86 mean [%]	RFI CD86 SD [%]	RFI CD54 mean [%]	RFI CD54 SD [%]	Viability rel. viability [%]	Viability SD of viability
**357**	96	16	88	16	**94**	1
**429**	116	13	97	17	**92**	3
**514**	121	30	75	3	**87**	5
**617**	109	6	83	2	**87**	1
**741**	103	24	70	9	**83**	5
**889**	95	-	66	16	**84**	2
**1067**	96	12	62	10	**79**	3
**1280**	101	17	62	10	**78**	4
**VC**	100	28	100	10	**100**	1
**LA 1000 µg/mL**	70	6	124	23	**99**	0
**DNCB 4 µg/mL**	**464**	127	**703**	127	**79**	3

(3) LEVASIL 200 40%

Results of DPRA with Levasil 200 40%

TableS7a: DPRA with Levasil 200 40%. Peak area, peptide concentration and peptide depletion of NC, PC and the test substance for cysteine-peptide.

Reaction with cysteine-peptide	peak area [mAU*s] at 220 nm			peptide concentration [mM]				
	sample 1	sample 2	sample 3	sample 1	sample 2	sample 3	mean	SD
NC: 0.05% BSA	492.2	492.4	494.0	0.508	0.509	0.510	0.509	0.001
Levasil 200 40%ig (ratio to peptide 1.7 : 1)	496.0	493.3	494.5	0.512	0.510	0.511	0.511	0.001
PC: EGDMA in 0.05% w/v BSA water	93.0	87.5	81.0	0.094	0.088	0.081	0.088	0.006

Reaction with cysteine-peptide	peptide depletion [%]				
	sample 1	sample 2	sample 3	mean	SD
NC: 0.05% BSA	0.14	0.10	-0.24	**0.00**	0.21
Levasil 200 40%ig (ratio to peptide 1.7 : 1)	-0.64	-0.08	-0.35	**-0.36**	0.28
PC: EGDMA in 0.05% w/v BSA water	81.57	82.69	84.00	**82.75**	1.22

TableS7b: DPRA with Levasil 200 40%. Peak area, peptide concentration and peptide depletion of NC, PC and the test substance for lysine-peptide.

Reaction with lysine-peptide	peak area [mAU*s] at 220 nm			peptide concentration [mM]				
	sample 1	sample 2	sample 3	sample 1	sample 2	sample 3	mean	SD
NC: 0.05% BSA	435.4	428.0	433.7	0.508	0.499	0.506	0.505	0.005
Levasil 200 40%ig (ratio to peptide 8.7 : 1)	431.0	434.9	428.1	0.503	0.508	0.499	0.503	0.004
PC: EGDMA in 0.05% w/v BSA water	383.6	374.3	381.4	0.447	0.436	0.445	0.443	0.006

Reaction with lysine-peptide	peptide depletion [%]				
	sample 1	sample 2	sample 3	mean	SD
NC: 0.05% BSA	-0.72	1.02	-0.30	**0.00**	0.91
Levasil 200 40%ig (ratio to peptide 8.7 : 1)	0.33	-0.59	1.00	**0.25**	0.80
PC: EGDMA in 0.05% w/v BSA water	11.35	13.50	11.86	**12.24**	1.13

Mean peptide depletion

TableS7c: DPRA with Levasil 200 40%. Mean peptide depletions of Cysteine, Lysine and both peptides.

	Cysteine-Peptide		Lysine-Peptide		
	**mean depletion [%]**	**SD [%]**	**mean depletion [%]**	**SD [%]**	**mean of both depletions [%]**
**Levasil 200 40%ig (ratio to peptide 1.7 : 1 or 8.7 : 1)**	-0.36	0.28	0.25	0.80	**0.12**
**PC: EGDMA in 0.05% w/v BSA water**	82.75	1.22	12.24	1.13	**47.49**

Results of LuSens with Levasil 200 40%

Preliminary cytotoxicity assessment

TableS8a: LuSens with Levasil 200 40%. Results of 1st preliminary cytotoxicity assessment. The CV75 value (= estimated concentration that affords 75% cell viability) was determined by linear regression from the concentration-response curve to be 67 µg/mL (corresponding to ca. 1801 µM).

Concentration (final test substance) [µM]	Concentration (final test substance) [µg/mL]	Concentration (test substance) [µM]	Concentration (test substance) [µg/mL]	mean $OD_{570-690}$ of 3 replicates	mean rel. viability [%]
VC	VC	VC	VC	0.585	**100**
1.1	0.07	4.3	0.16	0.583	**100**
2.2	0.13	8.7	0.32	0.588	**101**
11	0.65	43	1.6	0.612	**105**
22	1.3	87	3.2	0.655	**112**
108	6.5	433	16	0.620	**106**
217	13	866	32	0.597	**102**
1083	65	4330	160	0.010	2
2165	130	8660	320	0.003	0
4330	260	17320	640	0.001	0

TableS8b: LuSens with Levasil 200 40%. Results of 2nd preliminary cytotoxicity assessment. The CV75 value (= estimated concentration that affords 75% cell viability) was determined by linear regression from the concentration-response curve to be 56 µg/mL (corresponding to ca. 1503 µM).

Concentration (final test substance) [µM]	Concentration (final test substance) [µg/mL]	Concentration (test substance) [µM]	Concentration (test substance) [µg/mL]	mean $OD_{570-690}$ of 3 replicates	mean rel. Viability [%]
VC	VC	VC	VC	0.359	**100**
17	1.0	68	2.5	0.346	**97**
34	2.0	135	5.0	0.360	**100**
68	4.1	271	10	0.408	**114**
135	8.1	541	20	0.389	**108**
271	16	1083	40	0.385	**107**
541	33	2165	80	0.087	24
1083	65	4330	160	0.012	3
2165	130	8660	320	0.002	1
4330	260	17320	640	0.001	0

Main experiments

TableS8c: LuSens with Levasil 200 40%. Mean values and standard deviations of luciferase induction and rel. viability as well as p-values of t-test (experiment 1). Concentrations with fold inductions above 1.50 with rel. viability ≥70% and with statistical significance are indicated in bold and in grey when < 70% viability. The EC1.50 (the concentration resulting in a fold induction of 1.50) in the 1st experiment was calculated to be 33 µg/mL (corresponding to 554 µM).

**Concentration (test substance) [µg/mL]**	**Concentration (test substance) [µM]**	**1st experiment** fold induction		rel. viability [%]		t-test	
		**mean**	**SD**	**mean**	**SD**	**p-value**	**markers**
16	268	1.06	0.07	120	3	0.151	n.s.
19	321	1.20	0.10	123	5	0.030	*
23	386	1.24	0.08	114	6	0.005	**
28	463	1.27	0.10	131	9	0.011	*
**33**	**555**	**1.50**	**0.03**	**146**	**14**	**0.000**	**
**40**	**666**	**2.33**	**0.10**	**136**	**9**	**0.000**	**
**48**	**800**	**2.51**	**0.32**	**125**	**16**	**0.006**	**
**58**	**960**	**2.30**	**0.22**	**103**	**2**	**0.003**	**
VC	VC	1.00	0.14	100	8	-	-
**EGDMA 18 µg/mL**	**EGDMA 90.8 µM**	**5.08**	**0.90**	**91**	**3**	**0.000**	**
LA 450 µg/mL	LA 5000 µM	0.93	0.08	106	7	0.082	n.s.

TableS8d: LuSens with Levasil 200 40%. Mean values and standard deviations of luciferase induction and rel. viability as well as p-values of t-test (experiment 2). Concentrations with fold inductions above 1.50 with rel. viability ≥70% and with statistical significance are indicated in bold and in grey when < 70% viability. The EC1.50 (the concentration resulting in a fold induction of 1.50) in the 2nd experiment was calculated to be 33 µg/mL (corresponding to 557 µM).

**Concentration (test substance) [µg/mL]**	**Concentration (test substance) [µM]**	**2nd experiment** fold induction		rel. viability [%]		t-test	
		**mean**	**SD**	**mean**	**SD**	**p-value**	**markers**
16	268	1.12	0.15	111	1	0.158	n.s.
19	321	1.16	0.14	106	2	0.083	n.s.
23	386	1.14	0.04	108	5	0.001	**
28	463	1.19	0.08	118	7	0.016	*
**33**	**555**	**1.50**	**0.20**	**114**	**2**	**0.022**	*
**40**	**666**	**1.97**	**0.07**	**116**	**5**	**0.000**	**
**48**	**800**	**1.83**	**0.09**	**105**	**5**	**0.000**	**
**58**	**960**	**2.01**	**0.22**	**116**	**4**	**0.006**	**
VC	VC	1.00	0.12	100	4	-	-
**EGDMA 18 µg/mL**	**EGDMA 90.8 µM**	**4.88**	**0.40**	**87**	**2**	**0.000**	**
LA 450 µg/mL	LA 5000 µM	0.76	0.04	103	2	0.000	**

TableS8e: LuSens with Levasil 200 40%. Mean values and standard deviations of luciferase induction and rel. viability as well as p-values of t-test (experiment 3). Concentrations with fold inductions above 1.50 with rel. viability ≥70% and with statistical significance are indicated in bold and in grey when < 70% viability. The EC1.50 (the concentration resulting in a fold induction of 1.50) in the 3rd experiment was calculated to be 34 µg/mL (corresponding to 570 µM). Relative viability of all samples was comparable between standard measurement and when centrifuged.

Concentration (test substance) [µg/mL]	Concentration (test substance) [µM]	3rd experiment fold induction mean	SD	rel. viability [%] standard measurement mean	SD	rel. viability [%] centrifuged mean	SD	t-test p-value	markers
16	268	1.09	0.07	111	5	118	2	0.083	n.s.
19	321	1.08	0.08	108	2	116	2	0.096	n.s.
23	386	1.12	0.05	111	4	109	7	0.021	*
28	463	1.29	0.07	113	7	111	6	0.006	**
33	555	1.39	0.17	104	3	95	3	0.028	*
**40**	**666**	**2.19**	**0.09**	**104**	**0**	**107**	**10**	**0.000**	**
**48**	**800**	**2.45**	**0.35**	**103**	**3**	**100**	**7**	**0.009**	**
**58**	**960**	**2.77**	**0.32**	**104**	**3**	**103**	**7**	**0.005**	**
VC	VC	1.00	0.07	100	2	100	4	-	-
**EGDMA 18 µg/mL**	**EGDMA 90.8 µM**	**3.78**	**0.60**	**88**	**4**	**89**	**0**	**0.000**	**
LA 450 µg/mL	LA 5000 µM	0.86	0.09	99	3	93	2	0.010	*

TableS9f: LuSens with Levasil 200 40%. Mean values and standard deviations of luciferase induction and rel. viability as well as p-values of t-test (experiment 4). Concentrations with fold inductions above 1.50 with rel. viability ≥70% and with statistical significance are indicated in bold and in grey when < 70% viability.

Concentration (test substance) [µg/mL]	Concentration (test substance) [µM]	4th experiment fold induction mean	SD	rel. viability [%] standard measurement mean	SD	rel. viability [%] centrifuged mean	SD	t-test p-value	markers
16	268	0.94	0.06	115	2	112	7	0.113	n.s.
19	321	1.09	0.16	110	1	105	1	0.216	n.s.
23	386	1.24	0.14	107	0	107	5	0.036	*
28	463	1.07	0.06	111	4	105	2	0.100	n.s.
33	555	1.26	0.13	106	3	106	1	0.029	*
40	666	1.19	0.02	114	1	113	3	0.000	**
48	800	1.40	0.03	114	4	115	6	0.000	**
**58**	**960**	**2.23**	**0.16**	**100**	**3**	**103**	**2**	**0.001**	**
VC	VC	1.00	0.15	100	3	100	4	-	-
**EGDMA 18 µg/mL**	**EGDMA 90.8 µM**	**3.77**	**0.09**	**100**	**3**	**103**	**0**	**0.000**	**
LA 450 µg/mL	LA 5000 µM	0.82	0.05	98	2	101	6	0.000	**

Results of h-CLAT with Levasil 200 40%

Main experiments

TableS9a: h-CLAT with Levasil 200 40%. RFI CD86, RFI CD54 and rel. viability. Mean values and standard deviations of 1st experiment. RFI above 150% (CD86) or 200% (CD54) with rel. viability ≥50% are indicated in bold.

1st experiment - with densitiy gradient centrifugation							
Concentration (test substance) [µg/mL]	Concentration (final test substance) [µg/mL]	RFI CD86		RFI CD54		Viability	
		mean [%]	SD [%]	mean [%]	SD [%]	rel. viability [%]	SD of viability
**357**	**145**	74	2	165	5	**99**	0
**429**	**174**	66	5	150	11	**98**	0
**514**	**209**	63	4	151	25	**98**	0
**617**	**251**	57	11	150	4	**97**	0
**741**	**301**	70	26	175	9	**97**	0
**889**	**361**	87	1	195	7	**97**	1
**1067**	**433**	87	7	**201**	14	**97**	1
**1280**	**520**	70	11	194	22	**95**	1
**VC**		100	4	100	17	**100**	0
**LA 1000 µg/mL**		78	9	140	6	**100**	0
**DNCB 4 µg/mL**		**393**	65	**621**	220	**68**	8

TableS9b: h-CLAT with Levasil 200 40%. RFI CD86, RFI CD54 and rel. viability. Mean values and standard deviations of 2nd experiment. RFI above 150% (CD86) or 200% (CD54) with rel. viability ≥50% are indicated in bold.

2nd experiment - with with densitiy gradient centrifugation							
Concentration (test substance) [µg/mL]	Concentration (final test substance) [µg/mL]	RFI CD86		RFI CD54		Viability	
		mean [%]	SD [%]	mean [%]	SD [%]	rel. viability [%]	SD of viability
**357**	**145**	79	10	155	12	**100**	1
**429**	**174**	60	15	147	23	**99**	1
**514**	**209**	56	16	134	16	**97**	0
**617**	**251**	50	6	151	18	**97**	1
**741**	**301**	66	1	147	23	**95**	1
**889**	**361**	59	9	164	25	**93**	1
**1067**	**433**	51	1	141	19	**92**	2
**1280**	**520**	52	6	150	6	**92**	1
**VC**		100	21	100	10	**100**	1
**LA 1000 µg/mL**		79	2	120	24	**100**	1
**DNCB 4 µg/mL**		**275**	33	**469**	95	**58**	5

TableS9c: h-CLAT with Levasil 200 40%. RFI CD86, RFI CD54 and rel. viability. Mean values and standard deviations of 4th experiment. RFI above 150% (CD86) or 200% (CD54) with rel. viability ≥50% are indicated in bold.

4th experiment - with densitiy gradient centrifugation							
Concentration (test substance) [µg/mL]	Concentration (final test substance) [µg/mL]	RFI CD86		RFI CD54		Viability	
		mean [%]	SD [%]	mean [%]	SD [%]	rel. viability [%]	SD of viability
**357**	**145**	60	4	129	6	**100**	0
**429**	**174**	72	13	162	16	**100**	0
**514**	**209**	64	8	143	43	**99**	1
**617**	**251**	42	2	143	25	**99**	0
**741**	**301**	52	5	136	15	**99**	0
**889**	**361**	80	13	130	61	**98**	0
**1067**	**433**	50	7	142	6	**98**	2
**1280**	**520**	43	2	162	16	**98**	1
**VC**		100	16	100	5	**100**	1
**LA 1000 µg/mL**		67	12	138	18	**100**	0
**DNCB 4 µg/mL**		**235**	8	**501**	38	**84**	4

(4) SiO2 DQ 12

Results of DPRA with SiO2 DQ 12

Reaction with cysteine-peptide

TableS10a: DPRA with SiO2 DQ 12. Peak area, peptide concentration and peptide depletion of NC, PC and the test substance for cysteine-peptide.

Reaction with cysteine-peptide	peak area [mAU*min] at 220 nm			peptide concentration [mM]				
	sample 1	sample 2	sample 3	sample 1	sample 2	sample 3	mean	SD
**NC: 0.05% BSA**	7.62211	7.58030	7.40282	0.480	0.477	0.466	0.474	0.007
**DQ12 (ratio to peptide 4.3:1)**	7.49723	7.56322	7.59035	0.472	0.476	0.478	0.475	0.003
**DQ12 (ratio to peptide 10:1)**	7.42572	7.42915	7.20836	0.467	0.467	0.453	0.463	0.008
**PC: EGDMA in 0.05% w/v BSA water**	1.50004	2.03616	2.19041	0.091	0.125	0.135	0.117	0.023

Reaction with cysteine-peptide	peptide depletion [%]				
	sample 1	sample 2	sample 3	mean	SD
**NC: 0.05% BSA**	-1.17	-0.61	1.77	**0.00**	1.56
**DQ12 (ratio to peptide 4.3:1)**	0.51	-0.38	-0.74	**-0.20**	0.64
**DQ12 (ratio to peptide 10:1)**	1.47	1.42	4.38	**2.42**	1.69
**PC: EGDMA in 0.05% w/v BSA water**	80.86	73.68	71.61	**75.38**	4.85

Reaction with lysine-peptide

TableS10b: DPRA with SiO2 DQ 12. Peak area, peptide concentration and peptide depletion of NC, PC and the test substance for lysine-peptide.

Reaction with lysine-peptide	peak area [mAU*min] at 220 nm			peptide concentration [mM]				
	sample 1	sample 2	sample 3	sample 1	sample 2	sample 3	mean	SD
NC: 0.05% BSA	7.19465	7.23340	7.17578	0.498	0.500	0.496	0.498	0.002
DQ12 (ratio to peptide 21.3:1)	7.27004	7.26929	7.15124	0.503	0.503	0.495	0.500	0.005
PC: EGDMA in 0.05% w/v BSA water	6.30272	6.32587	6.33727	0.435	0.436	0.437	0.436	0.001

Reaction with lysine-peptide	peptide depletion [%]				
	sample 1	sample 2	sample 3	mean	SD
NC: 0.05% BSA	0.09	-0.45	0.36	**0.00**	0.42
DQ12 (ratio to peptide 21.3:1)	-0.97	-0.96	0.71	**-0.41**	0.97
PC: EGDMA in 0.05% w/v BSA water	12.70	12.37	12.21	**12.42**	0.25

Mean peptide depletion

TableS10c: DPRA with SiO2 DQ 12. Mean peptide depletions of Cysteine, Lysine and both peptides.

	Cysteine-Peptide		Lysine-Peptide		mean of both depletions [%]
	mean depletion [%]	SD [%]	mean depletion [%]	SD [%]	
**DQ12 (ratio to peptide 4.3:1 or 21.3:1)**	-0.20	0.64	-0.41	0.97	**0.00**
**DQ12 (ratio to peptide 10:1)**	2.42	1.69	-	-	**-**
**PC: EGDMA in 0.05% w/v BSA water**	75.38	4.85	12.42	0.25	**43.90**

Results of LuSens with SiO2 DQ 12

Preliminary cytotoxicity assessment

TableS11a: LuSens with SiO2 DQ 12. Results of preliminary cytotoxicity assessment. The CV75 value (= estimated concentration that affords 75% cell viability) was determined by linear regression from the concentration-response curve to be 498 µg/mL (corresponding to ca. 8286 µM).

Concentration (test substance) [µg/mL]	Concentration (test substance) [µM]	mean luminescence of 3 replicates	mean rel. viability [%]
VC	**VC**	4597762	**100**
29	**480**	4703000	**102**
35	**576**	4544661	**99**
42	**691**	4692913	**102**
50	**830**	4819774	**105**
60	**996**	4690977	**102**
72	**1195**	4732475	**103**
86	**1434**	4694767	**102**
103	**1720**	3989657	**87**
124	**2064**	4363110	**95**
149	**2477**	4693682	**102**
179	**2973**	4595306	**100**
214	**3567**	4491402	**98**
257	**4281**	4730405	**103**
309	**5137**	4126154	**90**
370	**6164**	4246156	**92**
444	**7397**	3936996	**86**
533	**8877**	3123796	68
640	**10652**	2857523	62

Main experiments

TableS11b: LuSens with SiO2 DQ 12. Mean values and standard deviations of luciferase induction and rel. viability as well as p-values of t-test (experiment 1). Concentrations with fold inductions above 1.50 with rel. viability ≥70% and with statistical significance are indicated in bold and in grey when < 70% viability.

Concentration (test substance) [µg/mL]	Concentration (test substance) [µM]	1st experiment fold induction mean	SD	rel. viability [%] mean	SD	t-test p-value	markers
**179**	**2973**	**2.05**	**0.09**	**93**	**7**	**0.000**	**
**214**	**3567**	**2.62**	**0.04**	**89**	**8**	**0.000**	**
**257**	**4281**	**3.26**	**0.19**	**93**	**3**	**0.001**	**
**309**	**5137**	**3.32**	**0.19**	**86**	**12**	**0.001**	**
**370**	**6164**	**3.15**	**0.08**	**86**	**3**	**0.000**	**
**444**	**7397**	**3.09**	**0.05**	**81**	**2**	**0.000**	**
**533**	**8877**	**3.08**	**0.20**	**87**	**6**	**0.001**	**
**640**	**10652**	**2.74**	**0.17**	**77**	**9**	**0.001**	**
VC	VC	1.00	0.09	100	9	-	-
**EGDMA 18 µg/mL**	**EGDMA 90.8 µM**	**3.72**	**0.48**	**89**	**11**	**0.000**	**
LA 450 µg/mL	LA 5000 µM	0.91	0.09	103	6	0.037	*

TableS11c: LuSens with SiO2 DQ 12. Mean values and standard deviations of luciferase induction and rel. viability as well as p-values of t-test (experiment 2). Concentrations with fold inductions above 1.50 with rel. viability ≥70% and with statistical significance are indicated in bold and in grey when < 70% viability.

Concentration (test substance) [µg/mL]	Concentration (test substance) [µM]	2nd experiment fold induction mean	SD	rel. viability [%] mean	SD	t-test p-value	markers
**179**	**2973**	**2.93**	**0.33**	**76**	**6**	**0.004**	**
**214**	**3567**	**1.51**	**0.13**	**91**	**5**	**0.007**	**
257	4281	1.17	0.22	90	9	0.159	n.s.
309	5137	1.31	0.07	86	4	0.003	**
**370**	**6164**	**2.05**	**0.44**	**83**	**12**	**0.026**	*
**444**	**7397**	**2.82**	**0.25**	**79**	**6**	**0.003**	**
**533**	**8877**	**2.50**	**0.36**	**74**	**9**	**0.009**	**
640	10652	2.22	0.24	65	5	0.006	**
VC	VC	1.00	0.10	100	6	-	-
**EGDMA 18 µg/mL**	**EGDMA 90.8 µM**	**4.69**	**0.44**	**85**	**10**	**0.000**	**
LA 450 µg/mL	LA 5000 µM	1.01	0.11	112	6	0.400	n.s.

Results of h-CLAT with SiO2 DQ 12

Main experiments

TableS12a: h-CLAT with SiO2 DQ 12. RFI CD86, RFI CD54 and rel. viability. Mean values and standard deviations of 1st experiment. RFI above 150% (CD86) or 200% (CD54) with rel. viability ≥50% are indicated in bold.

Concentration (test substance) [µg/mL]	1st experiment RFI CD86 mean [%]	RFI CD86 SD [%]	RFI CD54 mean [%]	RFI CD54 SD [%]	Viability rel. viability [%]	Viability SD of viability
**357**	115	19	**935**	544	**97**	1
**429**	117	37	**610**	71	**98**	1
**514**	113	29	**805**	332	**97**	1
**617**	62	38	**970**	354	**96**	0
**741**	109	25	**600**	141	**97**	0
**889**	110	13	**495**	177	**98**	1
**1067**	92	27	**530**	240	**97**	1
**1280**	72	28	**660**	198	**97**	1
**VC**	100	0	100	0	**100**	0
**LA 1000 µg/mL**	66	11	110	-	**100**	0
**DNCB 4 µg/mL**	**310**	54	**1061**	9	**81**	2

TableS12b: h-CLAT with SiO2 DQ 12. RFI CD86, RFI CD54 and rel. viability. Mean values and standard deviations of 2nd experiment. RFI above 150% (CD86) or 200% (CD54) with rel. viability ≥50% are indicated in bold.

Concentration (test substance) [µg/mL]	2nd experiment RFI CD86 mean [%]	RFI CD86 SD [%]	RFI CD54 mean [%]	RFI CD54 SD [%]	Viability rel. viability [%]	Viability SD of viability
**144**	**162**	44	**506**	70	**96**	1
**172**	**176**	35	**461**	8	**95**	2
**207**	**155**	24	**378**	63	**96**	4
**248**	138	38	**460**	152	**96**	2
**298**	**155**	32	**364**	3	**96**	3
**357**	148	44	**302**	3	**96**	1
**429**	127	16	**261**	8	**92**	4
**514**	140	43	**230**	83	**97**	0
**VC**	100	15	100	4	**100**	2
**LA 1000 µg/mL**	103	31	144	21	**96**	8
**DNCB 4 µg/mL**	**362**	92	**369**	42	**72**	10

(5) Z-COTE HP1

Results of DPRA with Z-Cote HP1

Reaction with cysteine-peptide

TableS13a: DPRA with Z-Cote HP1. Peak area, peptide concentration and peptide depletion of NC, PC and the test substance for cysteine-peptide.

	peak area [mAU*min] at 220 nm			peptide concentration [mM]				
**Reaction with cysteine-peptide**	**sample 1**	**sample 2**	**sample 3**	**sample 1**	**sample 2**	**sample 3**	**mean**	**SD**
**NC: 0.05% w/v BSA water**	7.74760	7.71554	7.41373	0.479	0.477	0.458	0.472	0.012
**Z-Cote HP1 (ratio to peptide 3.1:1)**	8.16896	8.11531	8.11344	0.506	0.502	0.502	0.504	0.002
**Z-Cote HP1 (ratio to peptide 10:1)**	8.10631	8.18191	7.92989	0.502	0.507	0.491	0.500	0.008
**PC: EGDMA in 0.05% w/v BSA water**	5.36238	5.23794	4.80425	0.329	0.321	0.294	0.315	0.018

	peptide depletion [%]				
**Reaction with cysteine-peptide**	**sample 1**	**sample 2**	**sample 3**	**mean**	**SD**
**NC: 0.05% w/v BSA water**	-1.63	-1.20	2.83	**0.00**	2.46
**Z-Cote HP1 (ratio to peptide 3.1:1)**	-7.25	-6.53	-6.51	**-6.76**	0.42
**Z-Cote HP1 (ratio to peptide 10:1)**	-6.41	-7.42	-4.06	**-5.96**	1.73
**PC: EGDMA in 0.05% w/v BSA water**	30.19	31.85	37.64	**33.23**	3.91

Reaction with lysine-peptide

Table S13b: DPRA with Z-Cote HP1. Peak area, peptide concentration and peptide depletion of NC, PC and the test substance for lysine-peptide.

	peak area [mAU*min] at 220 nm			peptide concentration [mM]				
**Reaction with lysine-peptide**	**sample 1**	**sample 2**	**sample 3**	**sample 1**	**sample 2**	**sample 3**	**mean**	**SD**
**NC: 0.05% w/v BSA water**	7.10453	7.07352	6.99380	0.490	0.488	0.483	0.487	0.004
**Z-Cote HP1 (ratio to peptide 15.7:1)**	7.21407	7.11719	7.48552	0.498	0.491	0.517	0.502	0.013
**PC: EGDMA in 0.05% w/v BSA water**	6.36949	6.39124	6.19867	0.439	0.440	0.427	0.435	0.007

	peptide depletion [%]				
**Reaction with lysine-peptide**	**sample 1**	**sample 2**	**sample 3**	**mean**	**SD**
**NC: 0.05% w/v BSA water**	-0.68	-0.23	0.91	**0.00**	0.82
**Z-Cote HP1 (ratio to peptide 15.7:1)**	-2.25	-0.86	-6.16	**-3.09**	2.75
**PC: EGDMA in 0.05% w/v BSA water**	9.89	9.58	12.35	**10.60**	1.52

Mean peptide depletion

TableS13c: DPRA with Z-Cote HP1. Mean peptide depletions of Cysteine, Lysine and both peptides.

	Cysteine-Peptide		Lysine-Peptide		
	mean depletion [%]	SD [%]	mean depletion [%]	SD [%]	mean of both depletions [%]
**Z-Cote HP1 (ratio to peptide 3.1:1 or 15.7:1)**	-6.76	0.42	-3.09	2.75	**0.00**
**Z-Cote HP1 (ratio to peptide 10:1)**	-5.96	1.73	-	-	**-**
**PC: EGDMA in 0.05% w/v BSA water**	33.23	3.91	10.60	1.52	**21.91**

Results of LuSens with Z-Cote HP1

Preliminary cytotoxicity assessment

TableS14a: LuSens with Z-Cote HP1. Results of 1st preliminary cytotoxicity assessment.

Concentration (test substance) [µg/mL]	Concentration (final test substance) [µM]	mean $OD_{570-690}$ of 3 replicates	mean rel. viability [%]
**VC**	**VC**	0.401	**100**
**2.5**	**30.7**	0.392	**98**
**5.0**	**61.4**	0.388	**97**
**10**	**123**	0.415	**104**
**20**	**246**	0.296	74
**40**	**492**	0.006	1
**80**	**983**	0.011	3
**160**	**1966**	0.023	6
**320**	**3932**	0.044	11
**640**	**7864**	0.087	22

TableS14b: LuSens with Z-Cote HP1. Results of 2nd preliminary cytotoxicity assessment. The CV75 value (= estimated concentration that affords 75% cell viability) was determined by linear regression from the concentration-response curve of the 2nd pre-test to be 13.7 µg/mL (corresponding to ca. 168 µM).

Concentration (test substance) [µg/mL]	Concentration (final test substance) [µM]	mean $OD_{570-690}$ of 3 replicates	mean rel. Viability [%]
**VC**	**VC**	0.385	**100**
**0.6**	**7.7**	0.402	**104**
**1.3**	**15.4**	0.387	**101**
**2.5**	**30.7**	0.436	**113**
**5.0**	**61.4**	0.437	**113**
**10**	**123**	0.443	**115**
**20**	**246**	0.025	7
**40**	**492**	0.007	2
**80**	**983**	0.019	5
**160**	**1966**	0.046	12

Main experiments

TableS14c: LuSens with Z-Cote HP1. Mean values and standard deviations of luciferase induction and rel. viability as well as p-values of t-test (experiment 1). Concentrations with fold inductions above 1.50 with rel. viability ≥70% and with statistical significance are indicated in bold and in grey when < 70% viability.

Concentration (test substance)		1st experiment fold induction		rel. viability [%]		t-test	
[µg/mL]	[µM]	mean	SD	mean	SD	p-value	markers
**7**	**82**	**1.63**	**0.12**	**101**	**2**	**0.004**	**
**8**	**99**	**2.24**	**0.25**	**98**	**1**	**0.006**	**
**10**	**119**	**2.14**	**0.43**	**101**	**3**	**0.022**	*
**12**	**142**	**2.42**	**0.26**	**88**	**2**	**0.005**	**
**14**	**171**	**3.72**	**0.08**	**95**	**3**	**0.000**	**
**17**	**205**	**5.04**	**0.54**	**86**	**5**	**0.003**	**
20	246	4.72	0.18	66	4	0.000	**
24	295	3.02	0.24	30	4	0.002	**
VC	VC	1.00	0.07	100	2	-	-
**EGDMA 18 µg/mL**	**EGDMA 90.8 µM**	**4.08**	**0.46**	**90**	**4**	**0.000**	**
LA 450 µg/mL	LA 5000 µM	0.90	0.08	92	3	0.017	*

TableS14d: LuSens with Z-Cote HP1. Mean values and standard deviations of luciferase induction and rel. viability as well as p-values of t-test (experiment 2). Concentrations with fold inductions above 1.50 with rel. viability ≥70% and with statistical significance are indicated in bold and in grey when < 70% viability.

Concentration (test substance)		2nd experiment fold induction		rel. viability [%]		t-test	
[µg/mL]	[µM]	mean	SD	mean	SD	p-value	markers
**7**	**82**	**2.21**	**0.13**	**113**	**3**	**0.001**	**
**8**	**99**	**2.40**	**0.07**	**105**	**3**	**0.000**	**
**10**	**119**	**2.86**	**0.06**	**107**	**1**	**0.000**	**
**12**	**142**	**3.41**	**0.30**	**104**	**1**	**0.002**	**
**14**	**171**	**5.17**	**1.09**	**92**	**3**	**0.011**	*
17	205	6.35	0.42	57	6	0.001	**
20	246	5.13	0.93	47	14	0.008	**
24	295	0.28	0.06	1	0	0.000	**
VC	VC	1.00	0.14	100	3	-	-
**EGDMA 18 µg/mL**	**EGDMA 90.8 µM**	**5.08**	**0.55**	**93**	**2**	**0.000**	**
LA 450 µg/mL	LA 5000 µM	0.85	0.06	104	4	0.001	**

Results of h-CLAT with Z-Cote HP1

Main experiments

TableS15a: h-CLAT with Z-Cote HP1. RFI CD86, RFI CD54 and rel. viability. Mean values and standard deviations of 1st experiment. RFI above 150% (CD86) or 200% (CD54) with rel. viability ≥50% are indicated in bold.

1st experiment						
**Concentration (test substance) [µg/mL]**	**RFI CD86**		**RFI CD54**		**Viability**	
	**mean [%]**	**SD [%]**	**mean [%]**	**SD [%]**	**rel. viability [%]**	**SD of viability**
**357**	121	31	**1888**	49	**76**	2
**429**	143	52	**2138**	196	**81**	2
**514**	**163**	49	**2585**	544	**80**	4
**617**	124	26	**1396**	81	**65**	7
**741**	143	48	**2495**	40	**73**	2
**889**	145	45	**2306**	20	**70**	3
**1067**	109	16	1954	762	49	14
**1280**	79	5	1472	558	30	13
**VC**	100	11	100	17	**100**	1
**LA 1000 µg/mL**	61	27	123	21	**100**	1
**DNCB 4 µg/mL**	**324**	86	**698**	146	**71**	5

TableS15b: h-CLAT with Z-Cote HP1. RFI CD86, RFI CD54 and rel. viability. Mean values and standard deviations of 2nd experiment. RFI above 150% (CD86) or 200% (CD54) with rel. viability ≥50% are indicated in bold.

2nd experiment						
**Concentration (test substance) [µg/mL]**	**RFI CD86**		**RFI CD54**		**Viability**	
	**mean [%]**	**SD [%]**	**mean [%]**	**SD [%]**	**rel. viability [%]**	**SD of viability**
**144**	90	14	**1413**	604	**86**	8
**172**	95	5	**1732**	55	**91**	3
**207**	86	10	**1324**	269	**85**	3
**248**	86	5	**1163**	193	**79**	2
**298**	68	6	**1144**	42	**81**	7
**357**	107	31	**1332**	338	**82**	4
**429**	79	14	**1221**	253	**80**	12
**514**	71	4	**1552**	822	**76**	19
**VC**	100	10	100	13	**100**	1
**LA 1000 µg/mL**	94	22	134	14	**99**	1
**DNCB 4 µg/mL**	**241**	20	**612**	87	**66**	6

(6) AEROSIL® R 972

Results of DPRA with AEROSIL® R 972

Reaction with cysteine-peptide

TableS16a: DPRA with AEROSIL® R 972. Peak area, peptide concentration and peptide depletion of NC, PC and the test substance for cysteine-peptide.

	peak area [mAU*min] at 220 nm			peptide concentration [mM]				
**Reaction with cysteine-peptide**	**sample 1**	**sample 2**	**sample 3**	**sample 1**	**sample 2**	**sample 3**	**mean**	**SD**
**NC: 0.05% BSA**	7.62211	7.58030	7.40282	0.480	0.477	0.466	0.474	0.007
**Aerosil R972 (ratio to peptide 4.3:1)**	7.70664	7.74654	7.60082	0.485	0.488	0.478	0.484	0.005
**Aerosil R972 (ratio to peptide 10:1)**	7.70855	7.69436	7.46062	0.485	0.484	0.469	0.480	0.009
**PC: EGDMA in 0.05% w/v BSA water**	1.50004	2.03616	2.19041	0.091	0.125	0.135	0.117	0.023

	peptide depletion [%]				
**Reaction with cysteine-peptide**	**sample 1**	**sample 2**	**sample 3**	**mean**	**SD**
**NC: 0.05% BSA**	-1.17	-0.61	1.77	**0.00**	1.56
**Aerosil R972 (ratio to peptide 4.3:1)**	-2.30	-2.83	-0.88	**-2.00**	1.01
**Aerosil R972 (ratio to peptide 10:1)**	-2.32	-2.13	1.00	**-1.15**	1.87
**PC: EGDMA in 0.05% w/v BSA water**	80.86	73.68	71.61	**75.38**	4.85

Reaction with lysine-peptide

TableS16b: DPRA with AEROSIL® R 972. Peak area, peptide concentration and peptide depletion of NC, PC and the test substance for lysine-peptide.

	peak area [mAU*min] at 220 nm			peptide concentration [mM]				
**Reaction with lysine-peptide**	**sample 1**	**sample 2**	**sample 3**	**sample 1**	**sample 2**	**sample 3**	**mean**	**SD**
**NC: 0.05% BSA**	7.19465	7.23340	7.17578	0.498	0.500	0.496	0.498	0.002
**Aerosil R972 (ratio to peptide 21.3:1)**	7.23453	7.26037	7.17305	0.500	0.502	0.496	0.500	0.003
**PC: EGDMA in 0.05% w/v BSA water**	6.30272	6.32587	6.33727	0.435	0.436	0.437	0.436	0.001

	peptide depletion [%]				
**Reaction with lysine-peptide**	**sample 1**	**sample 2**	**sample 3**	**mean**	**SD**
**NC: 0.05% BSA**	0.09	-0.45	0.36	**0.00**	0.42
**Aerosil R972 (ratio to peptide 21.3:1)**	-0.47	-0.83	0.40	**-0.30**	0.63
**PC: EGDMA in 0.05% w/v BSA water**	12.70	12.37	12.21	**12.42**	0.25

Mean peptide depletion

TableS16c: DPRA with AEROSIL® R 972. Mean peptide depletions of Cysteine, Lysine and both peptides.

	Cysteine-Peptide		Lysine-Peptide		mean of both depletions [%]
	mean depletion [%]	SD [%]	mean depletion [%]	SD [%]	
**Aerosil R972 (ratio to peptide 4.3:1 or 21.3:1)**	-2.00	1.01	-0.30	0.63	**0.00**
**Aerosil R972 (ratio to peptide 10:1)**	-1.15	1.87	-	-	**-**
**PC: EGDMA in 0.05% w/v BSA water**	75.38	4.85	12.42	0.25	**43.90**

Results of LuSens with AEROSIL® R 972

Preliminary cytotoxicity assessment

TableS17a: LuSens with AEROSIL® R 972. Results of preliminary cytotoxicity assessment. Calculation of a CV75 value (= estimated concentration that affords 75% cell viability) was not applicable.

Concentration (test substance) [µg/mL]	Concentration (test substance) [µM]	mean $OD_{570-690}$ of 3 replicates	mean rel. viability [%]
VC	**VC**	0.363	**100**
2.5	**42**	0.321	**88**
5	**83**	0.324	**89**
10	**166**	0.318	**88**
20	**333**	0.304	**84**
40	**666**	0.303	**83**
80	**1332**	0.387	**107**
160	**2663**	0.382	**105**
320	**5326**	0.350	**96**
640	**10653**	0.273	**75**

Main experiments

TableS17b: LuSens with AEROSIL® R 972. Mean values and standard deviations of luciferase induction and rel. viability as well as p-values of t-test (experiment 1). Concentrations with fold inductions above 1.50 with rel. viability ≥70% and with statistical significance are indicated in bold and in grey when < 70% viability.

**Concentration (test substance) [µg/mL]**	**Concentration (test substance) [µM]**	**1st experiment fold induction mean**	**SD**	**rel. viability [%] mean**	**SD**	**t-test p-value**	**markers**
179	2973	1.11	0.11	117	2	0.116	n.s.
214	3568	1.22	0.06	115	3	0.004	**
257	4281	1.22	0.05	128	6	0.001	**
**309**	**5137**	**1.79**	**0.29**	**121**	**0**	**0.020**	*
**370**	**6165**	**1.81**	**0.28**	**121**	**3**	**0.018**	*
**444**	**7398**	**1.64**	**0.14**	**125**	**8**	**0.005**	**
**533**	**8877**	**1.64**	**0.12**	**110**	**2**	**0.003**	**
**640**	**10653**	**1.72**	**0.09**	**108**	**4**	**0.001**	**
VC	VC	1.00	0.09	100	4	-	-
**EGDMA 18 µg/mL**	**EGDMA 90.8 µM**	**4.04**	**0.27**	**97**	**3**	**0.000**	**
LA 450 µg/mL	LA 5000 µM	0.87	0.05	100	2	0.001	**

TableS17c: LuSens with AEROSIL® R 972. Mean values and standard deviations of luciferase induction and rel. viability as well as p-values of t-test (experiment 2). Concentrations with fold inductions above 1.50 with rel. viability ≥70% and with statistical significance are indicated in bold and in grey when < 70% viability.

**Concentration (test substance) [µg/mL]**	**Concentration (test substance) [µM]**	**2nd experiment fold induction mean**	**SD**	**rel. viability [%] mean**	**SD**	**t-test p-value**	**markers**
179	2973	1.19	0.28	107	7	0.181	n.s.
214	3568	1.33	0.16	103	5	0.033	*
257	4281	1.32	0.04	102	4	0.000	**
309	5137	1.49	0.16	102	3	0.014	*
**370**	**6165**	**1.55**	**0.11**	**100**	**4**	**0.003**	**
444	7398	1.44	0.07	92	3	0.001	**
533	8877	1.48	0.15	93	3	0.012	*
640	10653	1.46	0.05	86	4	0.000	**
VC	VC	1.00	0.11	100	3	-	-
**EGDMA 18 µg/mL**	**EGDMA 90.8 µM**	**4.04**	**0.34**	**90**	**3**	**0.000**	**
LA 450 µg/mL	LA 5000 µM	0.96	0.08	97	3	0.182	n.s.

TableS17d: LuSens with AEROSIL® R 972. Mean values and standard deviations of luciferase induction and rel. viability as well as p-values of t-test (experiment 3). Concentrations with fold inductions above 1.50 with rel. viability ≥70% and with statistical significance are indicated in bold and in grey when < 70% viability.

Concentration (test substance) [µg/mL]	Concentration (test substance) [µM]	3rd experiment fold induction		rel. viability [%]		t-test	
		mean	SD	mean	SD	p-value	markers
179	2973	1.17	0.18	103	2	0.115	n.s.
214	3568	1.14	0.09	106	2	0.038	*
257	4281	1.18	0.09	108	2	0.025	*
309	5137	1.34	0.15	106	1	0.025	*
370	6165	1.45	0.17	104	2	0.018	*
444	7398	1.45	0.05	102	1	0.000	**
533	8877	1.40	0.09	108	4	0.003	**
**640**	**10653**	**1.50**	**0.12**	**99**	**6**	**0.006**	**
VC	VC	1.00	0.10	100	3	-	-
**EGDMA 18 µg/mL**	**EGDMA 90.8 µM**	**4.21**	**0.84**	**95**	**3**	**0.001**	**
LA 450 µg/mL	LA 5000 µM	0.96	0.03	101	2	0.096	n.s.

Results of h-CLAT with AEROSIL® R 972

Main experiments

TableS18a: h-CLAT with AEROSIL® R 972. RFI CD86, RFI CD54 and rel. viability. Mean values and standard deviations of 1st experiment. RFI above 150% (CD86) or 200% (CD54) with rel. viability ≥50% are indicated in bold.

1st experiment				
**Concentration (test substance) [µg/mL]**	**RFI CD86** [%]	**RFI CD54** [%]	**relative viability** mean [%]	SD [%]
**357**	107	100	**100**	0
**429**	107	100	**100**	0
**514**	93	100	**100**	0
**617**	80	80	**99**	0
**741**	117	120	**99**	0
**889**	101	90	**99**	0
**1067**	29	100	**99**	0
**1280**	109	110	**99**	0
**Controls:**				
**VC #1**	93	90	**100**	0
**VC #2**	107	110		
**LA 1000 µg/mL #1**	90	120	**100**	0
**LA 1000 µg/mL #2**	70	120		
**DNCB 4 µg/mL #1**	**237**	**377**	**93**	2
**DNCB 4 µg/mL #2**	**220**	**315**		

TableS18b: h-CLAT with AEROSIL® R 972. RFI CD86, RFI CD54 and rel. viability. Mean values and standard deviations of 2nd experiment. RFI above 150% (CD86) or 200% (CD54) with rel. viability ≥50% are indicated in bold.

2nd experiment				
**Concentration (test substance) [µg/mL]**	**RFI CD86** [%]	**RFI CD54** [%]	**relative viability** mean [%]	SD [%]
**357**	137	103	**100**	0
**429**	113	109	**100**	0
**514**	84	109	**99**	0
**617**	113	115	**99**	0
**741**	138	115	**99**	0
**889**	120	103	**99**	0
**1067**	107	103	**98**	0
**1280**	111	97	**98**	0
**Controls:**				
**VC #1**	92	91	**100**	1
**VC #2**	108	109		
**LA 1000 µg/mL #1**	75	121	**100**	0
**LA 1000 µg/mL #2**	71	115		
**DNCB 4 µg/mL #1**	**273**	**625**	**86**	2
**DNCB 4 µg/mL #2**	**245**	**538**		

(7) AEROSIL 200

Results of DPRA with AEROSIL 200

Reaction with cysteine-peptide

TableS19a: DPRA with AEROSIL 200. Peak area, peptide concentration and peptide depletion of NC, PC and the test substance for cysteine-peptide.

Reaction with cysteine-peptide	peak area [mAU*min] at 220 nm			peptide concentration [mM]				
	sample 1	sample 2	sample 3	sample 1	sample 2	sample 3	mean	SD
NC: 0.05% BSA	7.62211	7.58030	7.40282	0.480	0.477	0.466	0.474	0.007
Aerosil 200 (ratio to peptide 4.3:1)	7.71841	7.57555	7.50737	0.486	0.477	0.472	0.478	0.007
Aerosil 200 (ratio to peptide 10:1)	7.76771	7.50568	7.50781	0.489	0.472	0.472	0.478	0.010
PC: EGDMA in 0.05% w/v BSA water	1.50004	2.03616	2.19041	0.091	0.125	0.135	0.117	0.023

Reaction with cysteine-peptide	peptide depletion [%]				
	sample 1	sample 2	sample 3	mean	SD
NC: 0.05% BSA	-1.17	-0.61	1.77	**0.00**	1.56
Aerosil 200 (ratio to peptide 4.3:1)	-2.46	-0.54	0.37	**-0.88**	1.44
Aerosil 200 (ratio to peptide 10:1)	-3.12	0.39	0.37	**-0.79**	2.02
PC: EGDMA in 0.05% w/v BSA water	80.86	73.68	71.61	**75.38**	4.85

Reaction with lysine-peptide

TableS19b: DPRA with AEROSIL 200. Peak area, peptide concentration and peptide depletion of NC, PC and the test substance for lysine-peptide.

Reaction with lysine-peptide	peak area [mAU*min] at 220 nm			peptide concentration [mM]				
	sample 1	sample 2	sample 3	sample 1	sample 2	sample 3	mean	SD
NC: 0.05% BSA	7.19465	7.23340	7.17578	0.498	0.500	0.496	0.498	0.002
Aerosil 200 (ratio to peptide 21.3:1)	7.24698	7.25960	7.21425	0.501	0.502	0.499	0.501	0.002
PC: EGDMA in 0.05% w/v BSA water	6.30272	6.32587	6.33727	0.435	0.436	0.437	0.436	0.001

Reaction with lysine-peptide	peptide depletion [%]				
	sample 1	sample 2	sample 3	mean	SD
NC: 0.05% BSA	0.09	-0.45	0.36	**0.00**	0.42
Aerosil 200 (ratio to peptide 21.3:1)	-0.65	-0.82	-0.18	**-0.55**	0.33
PC: EGDMA in 0.05% w/v BSA water	12.70	12.37	12.21	**12.42**	0.25

Mean peptide depletion

TableS19c: DPRA with AEROSIL 200. Mean peptide depletions of Cysteine, Lysine and both peptides.

	Cysteine-Peptide		Lysine-Peptide		
	mean depletion [%]	SD [%]	mean depletion [%]	SD [%]	mean of both depletions [%]
Aerosil 200 (ratio to peptide 4.3:1 or 21.3:1)	-0.88	1.44	-0.55	0.33	**0.00**
Aerosil 200 (ratio to peptide 10:1)	-0.79	2.02	-	-	**-**
PC: EGDMA in 0.05% w/v BSA water	75.38	4.85	12.42	0.25	**43.90**

Results of LuSens with AEROSIL 200

Preliminary cytotoxicity assessment

TableS20a: LuSens with AEROSIL 200. Results of preliminary cytotoxicity assessment. The CV75 value (= estimated concentration that affords 75% cell viability) was calculated by linear regression to be 15 µg/mL or 243 µM.

Concentration (test substance) [µg/mL]	Concentration (test substance) [µM]	mean $OD_{570-690}$ of 3 replicates	mean rel. viability [%]
VC	**VC**	0.270	**100**
2.5	**42**	0.274	**101**
5	**83**	0.307	**114**
10	**166**	0.259	**96**
20	**333**	0.137	51
40	**666**	0.009	3
80	**1332**	0.000	0
160	**2663**	0.001	0
320	**5326**	0.000	0
640	**10653**	-0.001	0

Main experiments

TableS20b: LuSens with AEROSIL 200. Mean values and standard deviations of luciferase induction and rel. viability as well as p-values of t-test (experiment 1). Concentrations with fold inductions above 1.50 with rel. viability ≥70% and with statistical significance are indicated in bold and in grey when < 70% viability.

Concentration (test substance)	Concentration (test substance)	1st experiment - MTT centrifuged					
		fold induction		rel. viability [%]		t-test	
[µg/mL]	[µM]	mean	SD	mean	SD	p-value	markers
7	117	1.10	0.04	97	3	0.011	*
8	141	1.14	0.09	98	4	0.048	*
10	169	1.19	0.11	92	5	0.038	*
12	203	1.08	0.03	88	1	0.011	*
15	243	1.24	0.19	80	5	0.079	n.s.
18	292	0.85	0.10	74	10	0.054	n.s.
21	350	1.30	0.25	54	12	0.084	n.s.
25	420	0.95	0.17	42	4	0.329	n.s.
VC	VC	1.00	0.13	100	3	-	-
**EGDMA 18 µg/mL**	**EGDMA 90.8 µM**	**4.22**	**0.49**	**94**	**3**	**0.000**	**
LA 450 µg/mL	LA 5000 µM	1.00	0.19	99	2	0.499	n.s.

TableS20c: LuSens with AEROSIL 200. Mean values and standard deviations of luciferase induction and rel. viability as well as p-values of t-test (experiment 2). Concentrations with fold inductions above 1.50 with rel. viability ≥70% and with statistical significance are indicated in bold and in grey when < 70% viability.

Concentration (test substance) [µg/mL]	Concentration (test substance) [µM]	2nd experiment - MTT centrifuged fold induction mean	SD	rel. viability [%] mean	SD	t-test p-value	markers
7	117	1.20	0.15	108	5	0.064	n.s.
8	141	1.19	0.02	108	3	0.000	**
10	169	1.42	0.15	106	10	0.017	*
12	203	1.49	0.02	100	10	0.000	**
**15**	**243**	**1.69**	**0.17**	**98**	**3**	**0.007**	**
**18**	**292**	**1.85**	**0.04**	**93**	**9**	**0.000**	**
**21**	**350**	**1.64**	**0.06**	**73**	**12**	**0.000**	**
25	420	1.14	0.15	50	7	0.122	n.s.
VC	VC	1.00	0.10	100	4	-	-
**EGDMA 18 µg/mL**	**EGDMA 90.8 µM**	**4.79**	**0.50**	**87**	**3**	**0.000**	**
LA 450 µg/mL	LA 5000 µM	0.91	0.06	86	1	0.011	*

TableS20d: LuSens with AEROSIL 200. Mean values and standard deviations of luciferase induction and rel. viability as well as p-values of t-test (experiment 3). Concentrations with fold inductions above 1.50 with rel. viability ≥70% and with statistical significance are indicated in bold and in grey when < 70% viability.

Concentration (test substance) [µg/mL]	Concentration (test substance) [µM]	3rd experiment , MTT zentrifugiert fold induction mean	SD	rel. viability [%] mean	SD	t-test p-value	markers
7	117	1.02	0.06	102	4	0.308	n.s.
8	141	1.31	0.02	97	2	0.000	**
10	169	1.20	0.03	84	4	0.000	**
12	203	1.37	0.14	82	3	0.018	*
15	243	1.43	0.14	68	5	0.013	*
18	292	1.34	0.23	60	11	0.060	n.s.
21	350	0.96	0.22	25	3	0.399	n.s.
25	420	0.49	0.09	15	1	0.002	**
VC	VC	1.00	0.10	100	2	-	-
**EGDMA 18 µg/mL**	**EGDMA 90.8 µM**	**4.35**	**0.90**	**85**	**2**	**0.001**	**
LA 450 µg/mL	LA 5000 µM	0.90	0.06	87	3	0.009	**

Results of h-CLAT with AEROSIL 200

Main experiments

TableS21a: h-CLAT with AEROSIL 200. RFI CD86, RFI CD54 and rel. viability. Mean values and standard deviations of 1st experiment. RFI above 150% (CD86) or 200% (CD54) with rel. viability ≥50% are indicated in bold.

1st experiment				
**Concentration (test substance) [µg/mL]**	**RFI CD86** [%]	**RFI CD54** [%]	**relative viability** mean [%]	SD [%]
**357**	69	158	**99**	0
**429**	125	137	**99**	0
**514**	29	158	**99**	0
**617**	77	126	**99**	0
**741**	92	137	**98**	0
**889**	**171**	158	**97**	0
**1067**	73	168	**96**	1
**1280**	94	158	**92**	1
**Controls:**				
**VC #1**	81	74	**100**	0
**VC #2**	119	126		
**LA 1000 µg/mL #1**	119	126	**100**	0
**LA 1000 µg/mL #2**	131	105		
**DNCB 4 µg/mL #1**	**374**	**505**	**85**	4
**DNCB 4 µg/mL #2**	**361**	**463**		

TableS21b: h-CLAT with AEROSIL 200. RFI CD86, RFI CD54 and rel. viability. Mean values and standard deviations of 2nd experiment. RFI above 150% (CD86) or 200% (CD54) with rel. viability ≥50% are indicated in bold. RFI value for CD54 (429 µg/mL) and for CD86 and CD54 as well as relative viability (1280 µg/mL) are missing due to a technical error at sample preparation.

2nd experiment				
**Concentration (test substance) [µg/mL]**	**RFI CD86** [%]	**RFI CD54** [%]	**relative viability** mean [%]	SD [%]
**357**	78	164	**99**	0
**429**	78		**99**	0
**514**	65	154	**99**	0
**617**	79	138	**99**	0
**741**	63	128	**98**	0
**889**	68	169	**96**	1
**1067**	77	164	**94**	0
**1280**				
**Controls:**				
**VC #1**	87	82	**100**	0
**VC #2**	113	118		
**LA 1000 µg/mL #1**	65	97	**99**	0
**LA 1000 µg/mL #2**	68	87		
**DNCB 4 µg/mL #1**	**283**	**544**	**89**	1
**DNCB 4 µg/mL #2**	**255**	**506**		

TableS21c: h-CLAT with AEROSIL 200. RFI CD86, RFI CD54 and rel. viability. Mean values and standard deviations of 3rd experiment. RFI above 150% (CD86) or 200% (CD54) with rel. viability ≥50% are indicated in bold.

3rd experiment				
**Concentration (test substance) [µg/mL]**	**RFI CD86** [%]	**RFI CD54** [%]	**relative viability** mean [%]	SD [%]
**357**	63	**235**	**101**	0
**429**	73	**235**	**101**	0
**514**	60	141	**101**	0
**617**	59	165	**101**	0
**741**	61	153	**100**	0
**889**	70	141	**100**	0
**1067**	64	141	**99**	0
**1280**	53	188	**96**	1
**Controls:**				
**VC #1**	99	94	**100**	1
**VC #2**	101	106		
**LA 1000 µg/mL #1**	79	165	**99**	0
**LA 1000 µg/mL #2**	69	165		
**DNCB 4 µg/mL #1**	**235**	**645**	**81**	4
**DNCB 4 µg/mL #2**	**176**	**482**		

(8) TIO2 NIST® SRM®

Results of DPRA with TiO2 NIST® SRM®

Reaction with cysteine-peptide

TableS22a: DPRA with TiO2 NIST® SRM®. Peak area, peptide concentration and peptide depletion of NC, PC and the test substance for cysteine-peptide.

	peak area [mAU*min] at 220 nm			peptide concentration [mM]				
**Reaction with cysteine-peptide**	**sample 1**	**sample 2**	**sample 3**	**sample 1**	**sample 2**	**sample 3**	**mean**	**SD**
**NC: 0.05% w/v BSA water**	7.74760	7.71554	7.41373	0.479	0.477	0.458	0.472	0.012
**Titanium Dioxide (ratio to peptide 3.2:1)**	7.60493	7.66882	7.97965	0.470	0.474	0.494	0.480	0.013
**Titanium Dioxide (ratio to peptide 10:1)**	7.55005	7.78038	7.37947	0.467	0.481	0.456	0.468	0.013
**PC: EGDMA in 0.05% w/v BSA water**	5.36238	5.23794	4.80425	0.329	0.321	0.294	0.315	0.018

	peptide depletion [%]				
**Reaction with cysteine-peptide**	**sample 1**	**sample 2**	**sample 3**	**mean**	**SD**
**NC: 0.05% w/v BSA water**	-1.63	-1.20	2.83	**0.00**	2.46
**Titanium Dioxide (ratio to peptide 3.2:1)**	0.28	-0.58	-4.72	**-1.67**	2.67
**Titanium Dioxide (ratio to peptide 10:1)**	1.01	-2.06	3.28	**0.74**	2.68
**PC: EGDMA in 0.05% w/v BSA water**	30.19	31.85	37.64	**33.23**	3.91

Reaction with lysine-peptide

TableS22b: DPRA with TiO2 NIST® SRM®. Peak area, peptide concentration and peptide depletion of NC, PC and the test substance for lysine-peptide.

Reaction with lysine-peptide	peak area [mAU*min] at 220 nm			peptide concentration [mM]				
	sample 1	sample 2	sample 3	sample 1	sample 2	sample 3	mean	SD
NC: 0.05% w/v BSA water	7.10453	7.07352	6.99380	0.490	0.488	0.483	0.487	0.004
Titanium Dioxide (ratio to peptide 16:1)	7.20923	7.30617	7.20201	0.498	0.504	0.497	0.500	0.004
PC: EGDMA in 0.05% w/v BSA water	6.36949	6.39124	6.19867	0.439	0.440	0.427	0.435	0.007

Reaction with lysine-peptide	peptide depletion [%]				
	sample 1	sample 2	sample 3	mean	SD
NC: 0.05% w/v BSA water	-0.68	-0.23	0.91	**0.00**	0.82
Titanium Dioxide (ratio to peptide 16:1)	-2.19	-3.58	-2.08	**-2.62**	0.84
PC: EGDMA in 0.05% w/v BSA water	9.89	9.58	12.35	**10.60**	1.52

Mean peptide depletion

TableS22c: DPRA with TiO2 NIST® SRM®. Mean peptide depletions of Cysteine, Lysine and both peptides.

	Cysteine-Peptide		Lysine-Peptide		mean of both depletions [%]
	mean depletion [%]	SD [%]	mean depletion [%]	SD [%]	
Titanium Dioxide (ratio to peptide 3.2:1 or 16:1)	-1.67	2.67	-2.62	0.84	**0.00**
Titanium Dioxide (ratio to peptide 10:1)	0.74	2.68	-	-	**-**
PC: EGDMA in 0.05% w/v BSA water	33.23	3.91	10.60	1.52	**21.91**

Results of LuSens with TiO2 NIST® SRM®

Preliminary cytotoxicity assessment

TableS23a: LuSens with TiO2 NIST® SRM®. Results of preliminary cytotoxicity assessment. ATP assay was performed instead of MTT assay as implausible viability values were obtained with the MTT assay (not shown). However, a test for direct MTT-reduction appeared to be negative (no blue discoloration of MTT-solution after addition and incubation of the test substance). The CV75 value (= estimated concentration that affords 75% cell viability) was determined by linear regression from the concentration-response curve to be 351 µg/mL (corresponding to ca. 4398 µM).

Concentration (test substance) [µg/mL]	Concentration (test substance) [µM]	mean luminescence of 3 replicates	mean rel. viability [%]
VC	**VC**	5431031	**100**
29	**361**	6072069	**112**
35	**433**	5844707	**108**
42	**520**	5874885	**108**
50	**624**	5348403	**98**
60	**749**	5337693	**98**
72	**899**	5892077	**108**
86	**1078**	5051148	**93**
103	**1294**	4987509	**92**
124	**1553**	4214735	**78**
149	**1863**	5061582	**93**
179	**2236**	4860579	**89**
214	**2683**	4753080	**88**
257	**3220**	4268714	**79**
309	**3864**	4267496	**79**
370	**4637**	3986363	73
444	**5564**	3426641	63
533	**6677**	2901615	53
640	**8013**	2056758	38

Main experiments

TableS23b: LuSens with TiO2 NIST® SRM®. Mean values and standard deviations of luciferase induction and rel. viability as well as p-values of t-test (experiment 1). Concentrations with fold inductions above 1.50 with rel. viability ≥70% and with statistical significance are indicated in bold and in grey when < 70% viability.

Concentration (test substance) [µg/mL]	Concentration (test substance) [µM]	1st experiment, plate 2 fold induction mean	SD	rel. viability [%] mean	SD	t-test p-value	markers
42	520	0.76	0.04	93	11	0.000	**
50	624	0.81	0.11	92	3	0.038	*
60	749	0.75	0.08	84	6	0.004	**
72	899	0.79	0.14	88	2	0.053	n.s.
86	1078	0.69	0.09	84	3	0.004	**
103	1294	0.76	0.06	87	4	0.001	**
124	1553	0.75	0.11	82	4	0.019	*
149	1863	0.71	0.04	71	3	0.000	**
VC	VC	1.00	0.13	100	9	-	-
**EGDMA 18 µg/mL**	**EGDMA 90.8 µM**	**3.94**	**0.27**	**87**	**7**	**0.000**	**
LA 450 µg/mL	LA 5000 µM	0.94	0.19	96	3	0.282	n.s.

Concentration (test substance) [µg/mL]	Concentration (test substance) [µM]	1st experiment, plate 1 fold induction mean	SD	rel. viability [%] mean	SD	t-test p-value	markers
179	2236	0.63	0.13	76	1	0.015	*
214	2683	0.68	0.08	71	1	0.004	**
257	3220	0.62	0.07	67	4	0.002	**
309	3864	0.55	0.06	68	3	0.000	**
370	4637	0.62	0.05	56	2	0.000	**
444	5564	0.56	0.02	52	4	0.000	**
533	6677	0.48	0.04	48	2	0.000	**
640	8013	0.49	0.06	46	5	0.000	**
VC	VC	1.00	0.08	100	10	-	-
**EGDMA 18 µg/mL**	**EGDMA 90.8 µM**	**3.69**	**0.23**	**85**	**6**	**0.000**	**
LA 450 µg/mL	LA 5000 µM	0.82	0.06	91	7	0.000	**

TableS23c: LuSens with TiO2 NIST® SRM®. Mean values and standard deviations of luciferase induction and rel. viability as well as p-values of t-test (experiment 2). Concentrations with fold inductions above 1.50 with rel. viability ≥70% and with statistical significance are indicated in bold and in grey when < 70% viability.

Concentration (test substance) [µg/mL]	Concentration (test substance) [µM]	2nd experiment, plate 2 fold induction mean	SD	rel. viability [%] mean	SD	t-test p-value	markers
42	520	0.98	0.05	114	4	0.260	n.s.
50	624	1.01	0.06	113	5	0.395	n.s.
60	749	0.99	0.05	125	8	0.426	n.s.
72	899	0.80	0.09	110	9	0.026	*
86	1078	0.95	0.06	101	5	0.146	n.s.
103	1294	0.85	0.09	115	6	0.042	*
124	1553	0.74	0.01	110	2	0.000	**
149	1863	0.76	0.12	107	1	0.034	*
VC	VC	1.00	0.07	100	6	-	-
**EGDMA 18 µg/mL**	**EGDMA 90.8 µM**	**4.30**	**0.67**	**99**	**15**	**0.000**	**
LA 450 µg/mL	LA 5000 µM	1.08	0.07	118	9	0.026	*

Concentration (test substance) [µg/mL]	Concentration (test substance) [µM]	2nd experiment, plate 1 fold induction mean	SD	rel. viability [%] mean	SD	t-test p-value	markers
179	2236	0.88	0.08	88	5	0.046	*
214	2683	0.74	0.06	98	9	0.001	**
257	3220	0.70	0.03	98	8	0.000	**
309	3864	0.65	0.05	98	4	0.000	**
370	4637	0.84	0.21	85	11	0.162	n.s.
444	5564	0.81	0.05	72	3	0.002	**
533	6677	0.75	0.08	80	7	0.006	**
640	8013	0.70	0.08	62	1	0.004	**
VC	VC	1.00	0.11	100	9	-	-
**EGDMA 18 µg/mL**	**EGDMA 90.8 µM**	**4.81**	**0.45**	**104**	**8**	**0.000**	**
LA 450 µg/mL	LA 5000 µM	1.04	0.06	113	4	0.140	n.s.

Results of h-CLAT with TiO2 NIST® SRM®

Main experiments

TableS24a: h-CLAT with TiO2 NIST® SRM®. RFI CD86, RFI CD54 and rel. viability. Mean values and standard deviations of 1st experiment. RFI above 150% (CD86) or 200% (CD54) with rel. viability ≥50% are indicated in bold.

Concentration (test substance) [µg/mL]	RFI CD86		RFI CD54		Viability	
	**1st experiment**					
	mean [%]	SD [%]	mean [%]	SD [%]	rel. viability [%]	SD of viability
**357**	105	-	132	2	**88**	8
**429**	91	26	111	1	**87**	6
**514**	83	18	100	0	**80**	13
**617**	95	39	111	1	**77**	14
**741**	111	8	122	16	**60**	12
**889**	117	58	89	1	42	17
**1067**	108	58	116	9	41	19
**1280**	85	55	55	78	48	21
**VC**	100	22	100	19	**100**	0
**LA 1000 µg/mL**	73	20	143	17	**100**	0
**DNCB 4 µg/mL**	**285**	10	**521**	91	**88**	4

TableS24b: h-CLAT with TiO2 NIST® SRM®. RFI CD86, RFI CD54 and rel. viability. Mean values and standard deviations of 2nd experiment. RFI above 150% (CD86) or 200% (CD54) with rel. viability ≥50% are indicated in bold.

Concentration (test substance) [µg/mL]	RFI CD86		RFI CD54		Viability	
	**2nd experiment**					
	mean [%]	SD [%]	mean [%]	SD [%]	rel. viability [%]	SD of viability
**357**	105	13	113	4	**87**	1
**429**	125	15	93	18	**82**	4
**514**	100	0	103	4	**79**	5
**617**	128	58	88	39	**61**	8
**741**	126	32	103	33	**53**	4
**889**	112	5	72	12	34	10
**1067**	99	17	36	6	35	7
**1280**	98	18	124	48	31	10
**VC**	100	6	100	23	**100**	0
**LA 1000 µg/mL**	77	13	128	14	**100**	0
**DNCB 4 µg/mL**	**349**	46	**628**	165	**79**	9

(9) TITAN DIOXIDE P25

Results of DPRA with Titan dioxide P25

Reaction with cysteine-peptide

TableS25a: DPRA with Titan dioxide P25. Peak area, peptide concentration and peptide depletion of NC, PC and the test substance for cysteine-peptide.

	peak area [mAU*min] at 220 nm			peptide concentration [mM]				
Reaction with cysteine-peptide	sample 1	sample 2	sample 3	sample 1	sample 2	sample 3	mean	SD
NC: 0.05% w/v BSA water	7.74760	7.71554	7.41373	0.479	0.477	0.458	0.472	0.012
Titandioxid P25 (ungecoated) (ratio to peptide 3.2:1)	7.37540	7.74650	7.49432	0.456	0.479	0.463	0.466	0.012
Titandioxid P25 (ungecoated) (ratio to peptide 10:1)	7.57488	7.67884	7.64508	0.468	0.475	0.473	0.472	0.003
PC: EGDMA in 0.05% w/v BSA water	5.36238	5.23794	4.80425	0.329	0.321	0.294	0.315	0.018

	peptide depletion [%]				
Reaction with cysteine-peptide	sample 1	sample 2	sample 3	mean	SD
NC: 0.05% w/v BSA water	-1.63	-1.20	2.83	**0.00**	2.46
Titandioxid P25 (ungecoated) (ratio to peptide 3.2:1)	3.34	-1.61	1.75	**1.16**	2.53
Titandioxid P25 (ungecoated) (ratio to peptide 10:1)	0.68	-0.71	-0.26	**-0.10**	0.71
PC: EGDMA in 0.05% w/v BSA water	30.19	31.85	37.64	**33.23**	3.91

Reaction with lysine-peptide

TableS25b: DPRA with Titan dioxide P25. Peak area, peptide concentration and peptide depletion of NC, PC and the test substance for lysine-peptide.

	peak area [mAU*min] at 220 nm			peptide concentration [mM]				
Reaction with lysine-peptide	sample 1	sample 2	sample 3	sample 1	sample 2	sample 3	mean	SD
NC: 0.05% w/v BSA water	7.10453	7.07352	6.99380	0.490	0.488	0.483	0.487	0.004
Titandioxid P25 (ungecoated) (ratio to peptide 16:1)	7.23130	7.28516	7.26182	0.499	0.503	0.501	0.501	0.002
PC: EGDMA in 0.05% w/v BSA water	6.36949	6.39124	6.19867	0.439	0.440	0.427	0.435	0.007

	peptide depletion [%]				
Reaction with lysine-peptide	sample 1	sample 2	sample 3	mean	SD
NC: 0.05% w/v BSA water	-0.68	-0.23	0.91	**0.00**	0.82
Titandioxid P25 (ungecoated) (ratio to peptide 16:1)	-2.50	-3.28	-2.94	**-2.91**	0.39
PC: EGDMA in 0.05% w/v BSA water	9.89	9.58	12.35	**10.60**	1.52

Mean peptide depletion

TableS25c: DPRA with Titan dioxide P25. Mean peptide depletions of Cysteine, Lysine and both peptides.

	Cysteine-Peptide		Lysine-Peptide		
	**mean depletion [%]**	**SD [%]**	**mean depletion [%]**	**SD [%]**	**mean of both depletions [%]**
**Titandioxid P25 (ungecoated) (ratio to peptide 3.2:1 or 16:1)**	1.16	2.53	-2.91	0.39	**0.58**
**Titandioxid P25 (ungecoated) (ratio to peptide 10:1)**	-0.10	0.71	-	-	**-**
**PC: EGDMA in 0.05% w/v BSA water**	33.23	3.91	10.60	1.52	**21.91**

Results of LuSens with Titan dioxide P25

Preliminary cytotoxicity assessment

TableS26a: LuSens with Titan dioxide P25. Results of preliminary cytotoxicity assessment. The CV75 value (= estimated concentration that affords 75% cell viability) was determined by linear regression from the concentration-response curve to be 222 µg/mL (corresponding to ca. 2780 µM).

**Concentration (test substance) [µM]**	**Concentration (test substance) [µg/mL]**	**mean $OD_{570-690}$ of 3 replicates**	**mean rel. viability [%]**
VC	**VC**	0.431	**100**
31	**2.5**	0.386	**90**
63	**5.0**	0.395	**92**
125	**10**	0.381	**88**
250	**20**	0.434	**101**
501	**40**	0.414	**96**
1002	**80**	0.386	**90**
2003	**160**	0.364	**85**
4006	**320**	0.258	60
8013	**640**	0.192	44

Main experiments

TableS26b: LuSens with Titan dioxide P25. Mean values and standard deviations of luciferase induction and rel. viability as well as p-values of t-test (experiment 1). Concentrations with fold inductions above 1.50 with rel. viability ≥70% and with statistical significance are indicated in bold and in grey when < 70% viability.

Concentration (test substance)	Concentration (test substance)	1st experiment - MTT centrifuged					
		fold induction		rel. viability [%]		t-test	
[µg/mL]	[µM]	mean	SD	mean	SD	p-value	markers
103	1293	0.75	0.14	94	6	0.038	*
124	1551	0.89	0.08	101	3	0.066	n.s.
149	1862	0.83	0.10	92	5	0.042	*
178	2234	0.75	0.05	90	9	0.001	**
214	2681	0.78	0.14	72	2	0.056	n.s.
257	3217	0.72	0.07	66	1	0.003	**
308	3860	0.79	0.10	57	1	0.027	*
370	4632	0.87	0.06	56	2	0.021	*
VC	VC	1.00	0.10	100	4	-	-
**EGDMA 18 µg/mL**	**EGDMA 90.8 µM**	**5.51**	**0.53**	**100**	**2**	**0.000**	**
LA 450 µg/mL	LA 5000 µM	1.00	0.09	100	3	0.489	n.s.

TableS26c: LuSens with Titan dioxide P25. Mean values and standard deviations of luciferase induction and rel. viability as well as p-values of t-test (experiment 2). Concentrations with fold inductions above 1.50 with rel. viability ≥70% and with statistical significance are indicated in bold and in grey when < 70% viability.

Concentration (test substance)	Concentration (test substance)	2nd experiment - MTT centrifuged					
		fold induction		rel. viability [%]		t-test	
[µg/mL]	[µM]	mean	SD	mean	SD	p-value	markers
179	2236	0.52	0.08	70	7	0.001	**
214	2683	0.63	0.03	65	1	0.000	**
257	3220	0.66	0.03	64	2	0.000	**
309	3864	0.67	0.04	61	0	0.000	**
370	4637	0.67	0.01	57	2	0.000	**
444	5564	0.72	0.09	50	3	0.010	*
533	6677	0.66	0.04	44	1	0.000	**
640	8013	0.62	0.06	29	10	0.000	**
VC	VC	1.00	0.09	100	6	-	-
**EGDMA 18 µg/mL**	**EGDMA 90.8 µM**	**4.47**	**0.37**	**85**	**8**	**0.000**	**
LA 450 µg/mL	LA 5000 µM	0.80	0.07	102	7	0.000	**

TableS26d: LuSens with Titan dioxide P25. Mean values and standard deviations of luciferase induction and rel. viability as well as p-values of t-test (experiment 3). Concentrations with fold inductions above 1.50 with rel. viability ≥70% and with statistical significance are indicated in bold and in grey when < 70% viability.

Concentration (test substance) [µg/mL]	Concentration (test substance) [µM]	3rd experiment - centrifuged fold induction mean	SD	rel. viability [%] mean	SD	t-test p-value	markers
103	1293	0.59	0.02	79	0	0.000	**
124	1551	0.62	0.03	71	5	0.000	**
149	1862	0.55	0.00	77	3	0.000	**
179	2234	0.54	0.05	72	1	0.000	**
214	2681	0.62	0.06	60	11	0.000	**
257	3217	0.61	0.04	61	0	0.000	**
309	3860	0.73	0.07	58	3	0.003	**
370	4632	0.69	0.05	59	3	0.000	**
VC	VC	1.00	0.10	100	13	-	-
**EGDMA 18 µg/mL**	**EGDMA 90.8 µM**	**4.80**	**0.19**	**99**	**14**	**0.000**	**
LA 450 µg/mL	LA 5000 µM	0.85	0.10	97	11	0.013	*

Results of h-CLAT with Titan dioxide P25

Main experiments

TableS27a: h-CLAT with Titan dioxide P25. RFI CD86, RFI CD54 and rel. viability. Mean values and standard deviations of 1st experiment. RFI above 150% (CD86) or 200% (CD54) with rel. viability ≥50% are indicated in bold.

1st experiment				
Concentration (test substance) [µg/mL]	RFI CD86 [%]	RFI CD54 [%]	relative viability mean [%]	SD [%]
**357**	**157**	124	**73**	1
**429**	**200**	152	**67**	2
**514**	103	114	**57**	1
**617**	**166**	105	**54**	3
**741**	136	105	39	4
**889**	184	152	22	1
**1067**	133	114	20	5
**1280**	131	95	32	7
**Controls:**				
**VC #1**	109	86	**100**	0
**VC #2**	91	114		
**LA 1000 µg/mL #1**	89	105	**100**	0
**LA 1000 µg/mL #2**	136	124		
**DNCB 4 µg/mL #1**	**225**	**380**	**87**	2
**DNCB 4 µg/mL #2**	**224**	**400**		

TableS27b: h-CLAT with Titan dioxide P25. RFI CD86, RFI CD54 and rel. viability. Mean values and standard deviations of 2nd experiment. RFI above 150% (CD86) or 200% (CD54) with rel. viability ≥50% are indicated in bold.

2nd experiment				
**Concentration (test substance) [µg/mL]**	**RFI CD86** [%]	**RFI CD54** [%]	**relative viability** mean [%]	SD [%]
**357**	**156**	122	**92**	1
**429**	135	133	**89**	2
**514**	113	117	**85**	2
**617**	139	122	**79**	1
**741**	143	144	**71**	3
**889**	130	117	**76**	1
**1067**	139	139	**75**	4
**1280**	72	111	**65**	1
**Controls:**				
**VC #1**	106	94	**100**	0
**VC #2**	94	106		
**LA 1000 µg/mL #1**	93	94	**99**	0
**LA 1000 µg/mL #2**	82	94		
**DNCB 4 µg/mL #1**	**243**	**400**	**87**	2
**DNCB 4 µg/mL #2**	**222**	**417**		

TableS27c: h-CLAT with Titan dioxide P25. RFI CD86, RFI CD54 and rel. viability. Mean values and standard deviations of 3rd experiment. RFI above 150% (CD86) or 200% (CD54) with rel. viability ≥50% are indicated in bold.

3rd experiment				
**Concentration (test substance) [µg/mL]**	**RFI CD86** [%]	**RFI CD54** [%]	**relative viability** mean [%]	SD [%]
**357**	**213**	112	**73**	3
**429**	104	104	**66**	2
**514**	113	80	**51**	0
**617**	123	96	43	1
**741**	126	80	28	2
**889**	157	72	20	1
**1067**	122	72	19	1
**1280**	85	96	18	3
**Controls:**				
**VC #1**	120	96	**100**	1
**VC #2**	80	104		
**LA 1000 µg/mL #1**	58	128	**99**	0
**LA 1000 µg/mL #2**	53	112		
**DNCB 4 µg/mL #1**	**186**	**508**	**81**	5
**DNCB 4 µg/mL #2**	**184**	**442**		
